# Individual fates of mesenchymal stem cells *in vitro*

**DOI:** 10.1186/1752-0509-4-73

**Published:** 2010-05-27

**Authors:** Axel Krinner, Martin Hoffmann, Markus Loeffler, Dirk Drasdo, Joerg Galle

**Affiliations:** 1 Interdisciplinary Centre for Bioinformatics, University of Leipzig, Haertelstr. 16-18, 04107 Leipzig, Germany; 2 Institute for Medical Informatics, Statistics and Epidemiology, University of Leipzig, Haertelstr. 16-18, 04107 Leipzig, Germany; 3 French National Institute for Research in Computer Science and Control, Domaine de Voluceau-Rocquencourt, B.P. 105, 8153 Le Chesnay Cedex, France

## Abstract

**Background:**

*In vitro *cultivated stem cell populations are in general heterogeneous with respect to their expression of differentiation markers. In hematopoietic progenitor populations, this heterogeneity has been shown to regenerate within days from isolated subpopulations defined by high or low marker expression. This kind of plasticity has been suggested to be a fundamental feature of mesenchymal stem cells (MSCs) as well. Here, we study MSC plasticity on the level of individual cells applying a multi-scale computer model that is based on the concept of noise-driven stem cell differentiation.

**Results:**

By simulation studies, we provide detailed insight into the kinetics of MSC organisation. Monitoring the fates of individual cells in high and low oxygen culture, we calculated the average transition times of individual cells into stem cell and differentiated states. We predict that at low oxygen the heterogeneity of a MSC population with respect to differentiation regenerates from any selected subpopulation in about two days. At high oxygen, regeneration becomes substantially slowed down. Simulation results on the composition of the functional stem cell pool of MSC populations suggest that most of the cells that constitute this pool originate from more differentiated cells.

**Conclusions:**

Individual cell-based models are well-suited to provide quantitative predictions on essential features of the spatio-temporal organisation of MSC *in vitro*. Our predictions on MSC plasticity and its dependence on the environment motivate a number of *in vitro *experiments for validation. They may contribute to a better understanding of MSC organisation *in vitro*, including features of clonal expansion, environmental adaptation and stem cell ageing.

## Background

The generation and maintenance of replenishing tissues relies on an appropriately regulated balance between self-renewal and differentiation within a relatively small population of adult stem cells. According to the common stem cell paradigm this balance can be explained assuming a strict differentiation hierarchy and irreversible fate decisions [1,2]. However, the organisation of stem cell populations is strongly influenced by environmental factors such as specific cell-cell interactions, growth factor and oxygen supply, as well as the geometry and mechanical properties of the local environment [3,4]. Accordingly, it has been suggested that stemness represents a particular regulatory cell state rather than an entity and that this state may be approached in principle by any cell [5,6]. Supporting these ideas, recent experimental results in hematopoietic systems demonstrated that stem cell populations can actually regenerate from more differentiated subpopulations [7,8]. Currently, there is an ongoing debate on fundamental dynamics underlying this kind of cell plasticity. In particular, it remains open whether de-differentiation is prerequisite to lineage changes. A thorough understanding of this phenomenon is expected to make an important contribution to the development of novel therapeutic strategies for treating degenerative disease, injury and neoplasia.

Mesenchymal stem cells (MSCs) are multi-potent cells that persist in adult life in some tissue types, such as bone-marrow stroma, fat, skeletal muscle, and synovium without loosing their capacity to proliferate and differentiate [9,10]. Under appropriate culture conditions, they can multiply and transform into specialized cell types *in vitro*. Plasticity of MSCs of the 3T3 T type linked to de-differentiation has already been demonstrated in the Eighties [11]. More recently, also differentiation of adult human MSC was found to be at least partially reversible [12]. In fact plasticity has been suggested to represent a fundamental feature of MSC [13].

Recently, we have introduced a multi-scale computer model of MSC expansion, lineage commitment and differentiation which consistently explains a panel of experimental results regarding the oxygen dependence of these processes and predicts optimal culture conditions [14]. This model utilises the concept of noise-driven stem cell differentiation [15] which is based on the functional stem cell approach to tissue organisation by Roeder & Loeffler [5,16]. According to this concept, MSC plasticity bases on permanent fluctuations of the differentiation state of each individual cell, which enables more differentiated cells to re-gain stem cell properties and subsequently to switch lineage (details see below).

Here we aim at quantitative predictions on MSC organisation *in vitro *based on our former results. For this purpose we performed "experiments *in silico*" using our novel multi-scale model. We monitored the fates of individual MSCs under different culture conditions. Linking intracellular regulation of the differentiation state to cell biomechanics our computer simulations provide insight into possible mechanisms of how cell-cell and cell-substrate interaction can affect stem cell functionality. Thereby, our computer simulations were designed as MSC protocols *in silico *such that they can be directly tested *in vitro*.

In the following we first give a brief description of the model of MSC organisation *in vitro *introduced by Krinner et al. [14] and provide the experimentally validated data set used throughout this study. Subsequently, we present our simulation results on MSC plasticity and discuss the potential and the limits of our approach.

## Model

### Noise-driven differentiation dynamics

In our model cell differentiation is defined as the loss of stem cell properties. Cell differentiation is quantified by a continuous state variable α that can adopt values between zero (full stem cell competency) and one (completely differentiated cell). Each value of α may represent a set of regulatory network activation patterns. From the molecular point of view, α may depend on the abundance and sub-cellular localization of proteins and RNAs, as well as other types of signalling and metabolic molecules [17]. Cell differentiation is assumed to occur independently of cell proliferation [18].

**Figure 1:**
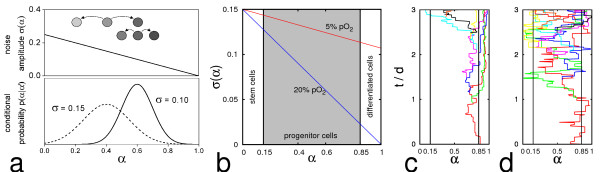
**Noise driven differentiation dynamics. **a) Modelling fluctuations of the differentiation state α. Upper panel: A decrease of the noise amplitude σ(α), i.e. the width of the Gaussian conditional probability function p(α|α'), with α results in an average drift to higher values of α. Lower panel: p(α|α') for α' = 0.4 and α' = 0.6. b) Noise amplitude for *in vitro *MSC expansion at 5% and 20% pO_2_. Proliferative states α_p _are shown in grey. c), d) Genealogies of expanding clones in the α-space of differentiation at 20% and at 5% pO_2_, respectively. α-trajectories of progeny arising from cell division are shown in individual colours.

The model assumes that each cell's α-value fluctuates randomly with a state dependent noise amplitude σ(α). From its current α value a cell adopts a new value α' with a transition rate R. α' is drawn from a Gaussian distribution p(α'| α), centred around α with standard deviation σ(α). According to this assumption, cells tend to accumulate in low noise states. The state dependence of σ(α) is further assumed to be determined by the environment. Hence, a differentiation-inducing environment reduces noise in high α states causing an accumulation of cells in differentiated states (see Figure 1a).

MSC differentiation involves lineage-priming [19]. This process implies particular cellular decisions, which can be modelled considering a second state variable [14]. We here assume that differentiation and de-differentiation dynamics do not depend on these decisions. However, a switch from one into another specific lineage may require a defined degree of stemness as suggested for the chondrogenic lineage [14]. In this case, differentiation stabilises lineages and the described capability of de-differentiation is synonymous with MSC plasticity in general. We here focus on that kind of MSC plasticity.

An important environmental factor during MSC expansion is oxygen [14,20]. In our model, we assume an oxygen dependent control of the state fluctuations. Increasing oxygen tension reduces the state fluctuations in differentiated states, thereby inducing unspecific differentiated, non-proliferative cells. This was implemented assuming the following dependence of the noise-amplitude σ(α) on the oxygen tension pO_2 _:(1)

where σ_0 _denotes the fluctuation strength in stem cell states and *f *is a Hill function approaching 0 and 1 at low and high pO_2_, respectively.

Cell proliferation is assumed to depend on the differentiation state α of a cell. In our model, it is restricted to intermediate differentiation states α_p _with: 0 < α_s _< α_p _< α_d _< 1 (Figure 1b). These states are termed 'progenitor states' in the following. For these proliferative states we assume an identical doubling rate r = 1/τ and average growth time τ. 'Stem cells' (α < α_s_) and 'differentiated cells' (α > α_d_) do not proliferate. The state fluctuations cause the cells to switch frequently between proliferative and non-proliferative state, which results in an effective average growth time larger than τ.

### Individual cell-based model (IBM)

In order to simulate the spatio-temporal dynamics of MSC populations we use an IBM where the cells are modelled as elastic adhesive spheres [21]. We assume that the cell volume in suspension cannot be smaller than a minimum value V_0_. A cell can move actively by migration and passively by being pushed, it can deform, adhere to other cells or a substrate, and it can grow and divide. A proliferating cell divides if its volume has grown to twice the volume V_0_.

Assuming that cells can approximately be described by an isotropic homogenous elastic solid, cell-cell and cell-substrate interaction are modelled by a modified Hertz-Potential, consisting of the classic Hertz-Potential and an adhesion term [22]:(2)

In the first term on the right hand side ν_i _denotes the Poisson's ratio of the interaction partner i (i = 1,2), E_i _its Young modulus, R_i _its radius (substrate radius R = ∞) and δ the surface deformation. The second term models adhesion proportional to the Hertz contact area, where ε_12 _is the anchorage given as adhesion energy per unit area.

Cell proliferation is modelled assuming a two phase cell cycle: During the interphase, a cell doubles its volume by stochastic increments. During the mitotic phase, a cell divides into two daughter cells of equal volume. This growth process results in an approximately Γ-distributed growth time τ of the cells [23]. A cell undergoes a growth arrest if the sum of the deformation forces on it exceeds a critical value F_c_.

We simulate cell motion by using a Langevin equation for each cell [21]. The small Reynolds numbers in the regime of single cells allows us to neglect inertia, leading to a linear system of stochastic equations for the cell displacements. Thereby, the displacement d**x**_i _of cell i is given by:(3)

where the sums run over all neighbouring cells j in direct contact to cell i. **F**_ij_^Hertz ^denotes the Hertz force between cell i and cell j and F_i_^stoch ^the stochastic Langevin force on cell i. The friction coefficients γ_is _and γ_ij _describe friction between cell i and the substrate and between cell i and cell j, respectively. These coefficients are assumed to be proportional to the respective contact areas. Details can be found in [14].

### Master equation approach

In addition to the IBM we pursue a theoretical population dynamics model as previously described [15]. Here, we use this model for studying the population average of dynamic properties of individual cells; therefore proliferation is not included. The model is then equivalent to a master equation for a Markov process [24] describing the dynamics of the average number of cells N(α) in state α:(5)

with transition probability  and constant randomization rate R. Transition times ϑ(α) from an initial α into the regimes of stem cells (α < α_s_) or differentiated cells (α > α_d_) were computed using an absorbing boundary approach [24].

### Model parameters

Our model of MSC differentiation dynamics depends on parameters describing intracellular regulation; the randomization rate R, the stem cell state fluctuation strength σ_0_, the parameters of the Hill function (n and k) and those specifying the proliferation rate (r and α_s _with α_d _= 1-α_s_). The IBM of spatio-temporal organisation of growing MSC populations depends on parameters specifying cell-cell and cell-substrate interaction, as the Poisson's ratio, the Young modulus, and the friction constants. Combining these models in a particular application one has to adjust a large parameter set.

Recently, we have applied the combined multi-scale model to ovine MSC expansion at low (5%) and high (20%) oxygen tension [14]. These former investigations enable us to use an experimentally validated set of model parameters in the present study. These parameters are summarized in Table 1. We used this parameter set in all simulations if not further specified.

**Table 1:** Parameter set used in the simulations

Parameter	Value
**Intracellular regulation**	
Randomization Rate R	2.5 × 10^4 ^s^-1^
Stem Cell State Fluctuation Strength σ_0_	0.15
Hill Coefficient n	5
Dissociation Constant k	0.3
Differentiation Threshold α_s _(α_d _= 1-α_s_)	0.85

**Spatio-temporal organisation**	
Minimal Cell Radius R_0_	5 μm
Minimal Cell Volume V_0_	V(R_0_)
Proliferation Rate r = 1/τ	1.9/day
Young Modulus E	450 Pa
Contact Inhibition Threshold F_max_	1 × 10^-9 ^N
Poisson's Ratio ν	0.4
Friction Coefficients γ_ij, _γ_is_	3 × 10^7 ^Ns/m^3 ^, 1 × 10^11 ^Ns/m^3 ^*
Cellular Diffusion Coefficient D_Cell_	4 × 10^-12 ^cm^2^/s
Cell-Cell Anchorage ε	6 × 10^-5 ^N/m
Cell-Plane Anchorage ε	6 × 10^-5 ^N/m
Quiescence Threshold F_q_	10 N/m^2^

The parameter set was adjusted using experimental data on the clone size distribution of ovine MSC growing *in vitro *[14]. ^* ^The high substrate friction coefficient γ_is _was used in 'population regeneration' simulations (Sec. 3.2) in order to study the influence of biophysical properties on stem cell plasticity.

## Results

### Monitoring individual cell fates

Using the IBM the fates of individual cells in growing populations can be monitored. We simulated individual α-trajectories and compared the cell differentiation dynamics at low (5%) and high (20%) oxygen concentrations. The genealogies of two selected clones in α space are shown in Figure 1c and 1d, for low and high oxygen, respectively.

**Figure 2:**
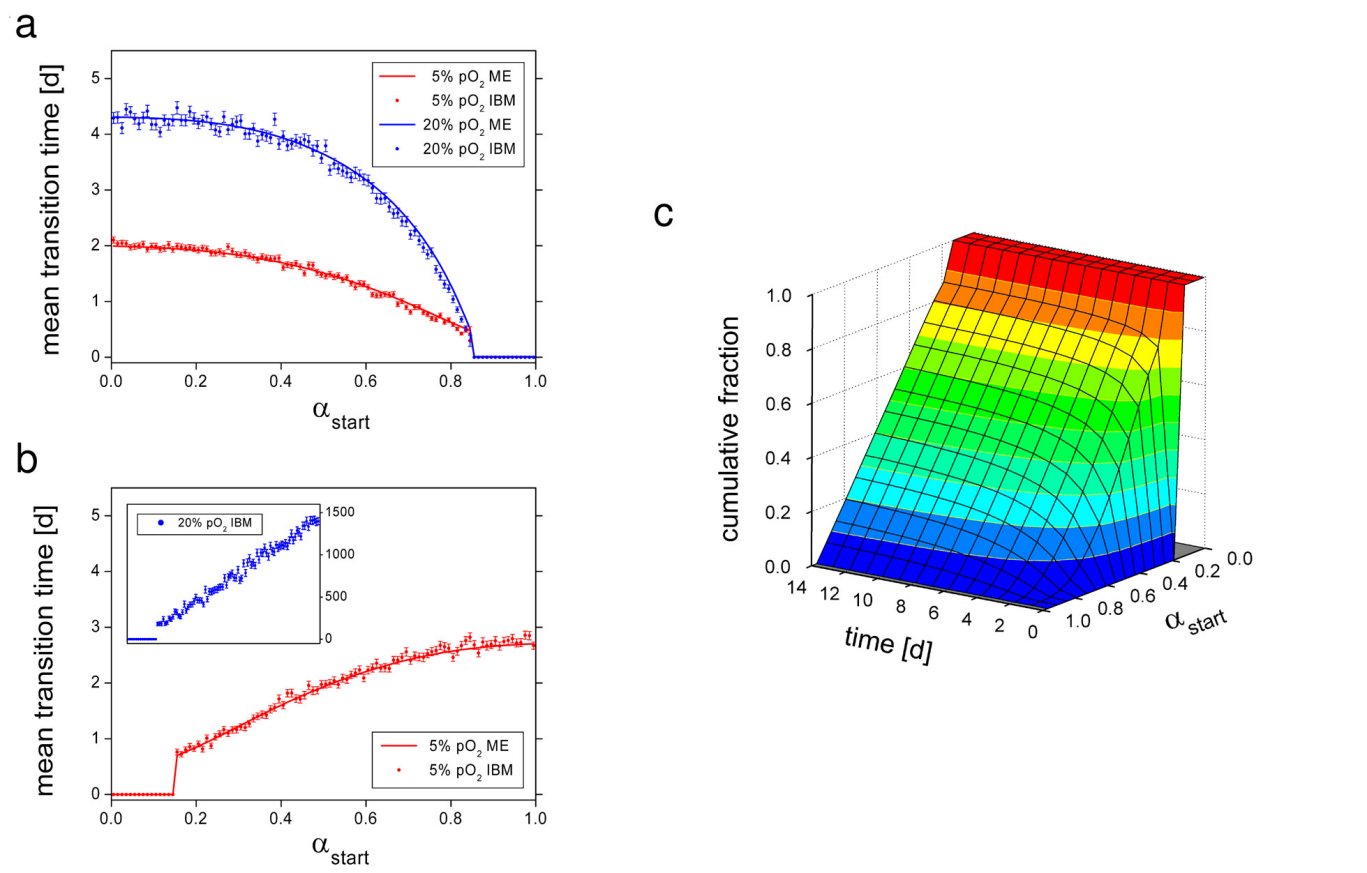
**Simulated individual cell dynamics. **a), b) Mean transition times calculated using IBM and the master equation approach (ME) to reach (a) differentiated states and (b) stem cell states at 5% (red) and at 20% pO_2 _(blue). The transition time to stem cell states at 20% pO_2 _was not calculated using the ME because the fraction of cells that have reached stem cell states once converged too slowly (see c). Symbols: IBM results, Lines: Master equation. (c) Fraction of cells that reach stem cell states at 20% pO_2 _(ME) as a function of initial α and simulation time.

In order to quantify the degree of plasticity that is inherent in MSCs we calculated the average time required to adopt specific cellular phenotypes. The average transition times of a cell to reach stem cell states (0.0 < α < α_s _= 0.15) and differentiated states (α_d _= 0.85 < α < 1.0) were calculated as follows: 100.000 cells with α-values equally distributed in the interval [0,1] were subjected to state fluctuations. Throughout the simulations cells that reached the specified subpopulation for the first time were counted and histograms about their initial state were derived. From these histograms we calculated the i) average transition times (Figure 2a, b) and ii) the fractions of cells that successfully transferred within a defined time.

Our results demonstrate that at low oxygen a frequent exchange between the subpopulations occurs on a time scale of about 2 days. At high oxygen the average transition time for stem cells into the pool of differentiated cells increases to about 4 days. Transition times for differentiated cells into the stem cell pool at high oxygen are much larger (>100 days), indicating quasi-deterministic cell differentiation behaviour. We confirmed our results using the master equation approach. In Figure 2c the fraction of cells having entered the stem cell pool at 20% pO_2 _is shown as a function of the initial α value and the simulation time. Only in this particular case, the fraction of absorbed cells grows too slowly to calculate the average transition times. In the three other cases, they were computed with high precision (less than 10^-12 ^of all cells remain to be absorbed).

Since stem cell states are more easily accessible at low oxygen compared to high oxygen we predict MSC plasticity to be more pronounced under these conditions.

*In vitro *validation of the above results would require single cell tracking of MSCs and techniques to identify the differentiation state of the tracked cells. Currently, considerable effort is taken in order to establish tracking techniques for stem cell systems [25,26]. Unfortunately, MSCs are particularly hard to track, because they tend to aggregate; a phenomenon known as mesenchymal condensation [27,28]. Thus, in the following we present results on MSC plasticity as seen on the population level which can be validated in simpler experimental setups.

### Modelling regeneration of the population structure

Chang et al. [7] studied how fast the distribution of differentiation marker expression within a cell population regenerates from subpopulations with defined expression level. They performed the following experiment: a population of precursor cells was generated under standard conditions and characterised by the expression level of a particular differentiation marker. Subpopulations of cells with defined expression levels of the differentiation marker were separated. These subpopulations were cultivated under standard conditions and regeneration of the distribution of expression levels in the population was monitored over time by FACS.

**Figure 3:**
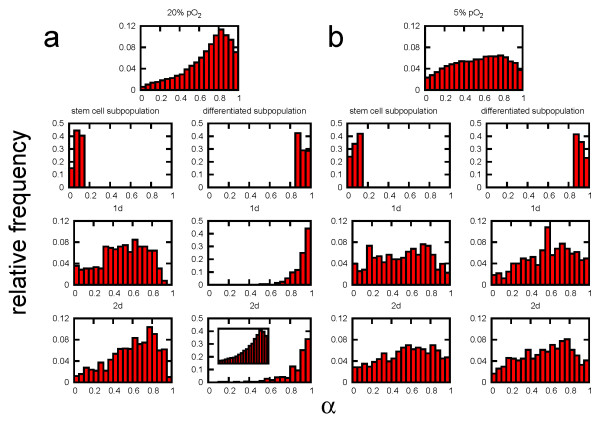
**Simulated regeneration of the population structure. **Shown are results for a representative regeneration simulation (a) at 20% pO_2 _and (b) at 5% pO_2_. For each oxygen concentration the regeneration from stem cells (left) is compared to regeneration from differentiated cells (right). The insert shows the regenerated population structure after 8d.

We simulated this population regeneration experiment as follows: Starting from a population that was grown at low density, i.e. which shows no signs of contact inhibition of growth, we selected 200 stem cells and 200 differentiated cells and followed their development over 5 days in secondary cultures. In order to characterise the environmental dependence of the regeneration process, we compared the MSC behaviour at low and high oxygen tension. Figure 3 shows the results for a selected realisation.

**Figure 4:**
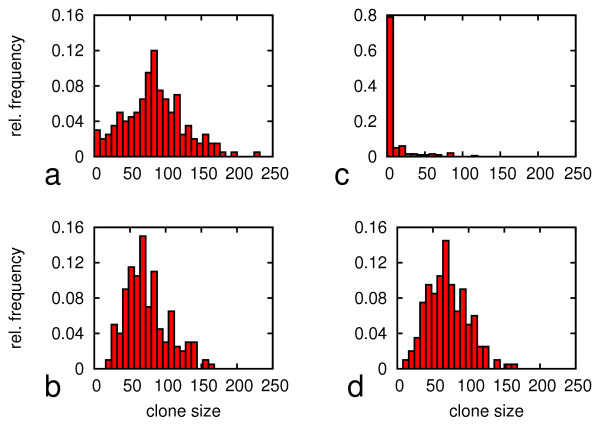
**Simulated clonal development during the regeneration. **Shown are the size distributions of 200 clones grown from stem cells (a,b) and differentiated cells (c,d) after 5 days of secondary culture. Upper row: 20% pO_2_. Lower row: 5% pO_2_.

At low oxygen the population structure is roughly regenerated by stem cells and by differentiated cells within about 1 day. At high oxygen the population is regenerated in about 2 days by stem cells but it takes about 8 days when starting with differentiated cells. This is still a surprisingly short time taking into account the large transition times for differentiated cells into the stem cell pool. This phenomenon can be understood by analysing the clone sizes of the 200 selected clones. The distributions of clone sizes after 5 days for all considered cases are shown in Figure 4. Except for regeneration from a differentiated subpopulation at high oxygen the distribution peak is located at about 50-100 cells per clone, demonstrating that most of the clones started growing. If regeneration started from differentiated cells at high oxygen, most of the cells remained quiescent throughout the observation time (137 out of 200 in Figure 4c) and only a few cells started to proliferate and formed large clones. This means the regeneration is driven by the progeny of these few cells only.

### Linking biomechanics and differentiation

**Figure 5:**
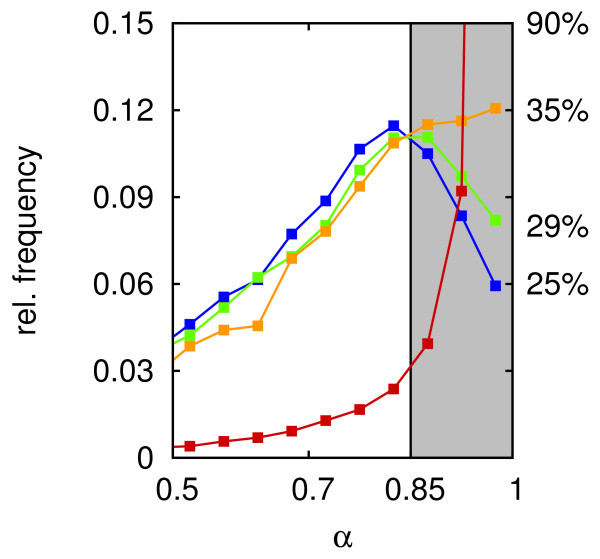
**Impact of contact inhibition of growth on regeneration. **The Figure shows the equilibrium distributions of differentiation states without contact inhibition of growth (blue) and without proliferation (red) at 20% pO_2_. For comparison two additional distributions obtained for populations with weak (green) and strong (yellow) contact inhibition are shown. On the right the fraction of cells in differentiated states (α > α_d_) is given. These distributions refer to regeneration experiments from differentiated cells after 7.5 days.

At the centre of expanding MSC clones proliferation becomes contact inhibited. The quiescent region grows with colony size until all cells will stop proliferation, when an expanding *in vitro *culture becomes confluent. Such changes in proliferation activity affect the population structure of MSC colonies. Figure 5 compares the α-distributions of different MSC populations at high oxygen (20% pO_2_). Shown are the α-distributions in a low-density population without any sign of contact inhibition, in growing clones with weak and strong contact inhibition induced by variation of the cell-substrate friction constants and in a confluent and thus quiescent population. The fraction of differentiated, non-proliferative cells (α > α_d_) increases from about 25% in the low density population to about 90% in the confluent population. A comparable induction of spontaneous differentiation in MSC can be observed *in vitro *(per. communication, A. Stolzing).

These simulation results implicate that if regeneration refers to the growth of a few large clones, as in the case of differentiated cells at high oxygen, the effect of contact inhibition becomes more relevant for population regeneration. The α-distribution in large clones significantly differs from that of a low-density culture. Moreover, due to the increased number of differentiated cells, these populations show a lower CFU capacity (compare [14]).

### Modelling the organisation of the stem cell pool

In general, 'self-renewal' of the stem cell population appears in our model as steady occupancy of stem cell states due to a particular population dynamics. Thus, additional information on MSC organisation *in vitro *can be obtained by performing the regeneration experiments described above in parallel for all subpopulations. Splitting the mother population into a number of subpopulations according to the expression of a differentiation marker, applying the 'regeneration protocol' suggested above to each of these subpopulations and quantifying the number of stem cells in each subpopulation after a fixed regeneration time would allow to quantify the fraction of stem cells in a MSC population descending from a particular subpopulation.

**Figure 6:**
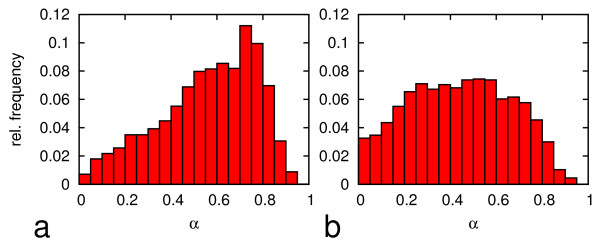
**Simulated clonal competition in the stem cell pool. **The histograms display the simulation results for the clonal composition of the stem cell pool (0 < α < α_s _= 0.15) for populations that were expanded at (a) 20% and (b) 5% pO_2 _for 5 days.

In additional simulations, we followed this concept. However, instead of splitting the mother population into subpopulations, we separated each individual cell of the mother population and followed expansion of the clones generated by the individual cells. For different time points we quantified the clonal composition of the common stem cell pool (0 < α < α_s _= 0.15) of all clones in terms of the initial α values of the cells that induced the clones. Figure 6 shows this clonal composition of the stem cell pool after 5 days of clonal expansion. At low oxygen (5% pO_2_) the fraction of stem cells that originate from stem cells is about 11%. At high oxygen (20% pO_2_) this fraction decreases to only 5%. In both cases, most of the cells in the stem cell pool originate from progenitor states. At low oxygen tension, all progenitor states equally contribute to this pool, while at high oxygen tension most cells originate from progenitor states with a high α value between 0.7 and 0.8.

## Discussion

Recent experimental findings indicate that cells can regain stem cell properties under defined environmental conditions. These results challenge the commonly agreed stem cell paradigm. This paradigm treats 'stemness' as a fixed property intrinsic to stem cells and assumes a deterministic and irreversible differentiation scenario for each cell [29]. As an alternative, novel concepts of functional stem cells have been developed that assign the interaction between cells and their growth environment a greater emphasis [5,13,16]. Treating stemness no longer as a fixed property, these concepts do not exclude certain preferred trends in the differentiation sequence, but allow reversible developments for individual cells.

We here provided the first quantitative predictions on the environmental dependent organisation of MSCs *in vitro *applying this novel concept. We predicted: i) the average transition times of individual cells into stem cell and differentiated states, ii) the time scales of the regeneration of the distribution of differentiation marker expression in a MSC population from subpopulations of stem and unspecific differentiated cells, and iii) the origin of the cells forming the *in vitro *stem cell pool of MSC. Moreover, we predicted that all these properties depend on the environment. Our results also provide estimates of the time scales of MSC adaptation to changed environmental conditions. They are in good agreement with experimental findings on MSC adaptation to low oxygen [30-32]. Particularly the work of Tang et al. [32] and Volkmer et al. [31] strengthens our modelling approach because the experimentally observed improvement of the functional competence of an entire MSC population within less than 24 hours can only hardly be explained by the expansion of residual stem cells as suggested by pedigree models.

In all our simulations, we considered an oxygen dependence of the state fluctuations.

In contrast, biophysical features, as cell-cell and cell-substrate interactions, were assumed to affect the regenerative potential of the MSC by interfering with their proliferation control mechanisms only. A direct feedback of these interactions on the noise amplitudes was not considered. However, recent results demonstrate that lineage specification and proliferation of MSC populations can be triggered by substrate elasticity [33] and substrate micro-structure [34]. Thus, we here suggest performing the proposed experiments on MSC plasticity on substrates that vary with respect to their elasticity and microstructure. These experiments would provide information on whether mechano-signalling can affect the kinetics of state transitions in MSCs and thus, can be used to time regeneration processes *in vitro*.

Our results on the composition of the stem cells pool suggest that most of the stem cells in MSC populations expanding *in vitro *originate from progenitor states. Thus, their mother cells underwent differentiation and de-differentiation processes and were proliferative active. Recent experimental results suggest that these cellular activities result in changes in the cellular phenotype called stem cell ageing [35]. A model that consistently describes these phenomena is currently lacking.

Most of our results could be validated by *in vitro *experiments on the population level. A number of suggestions were given in the text. However, more detailed studies would require tracking of individual cell fates in a single expanding MSC population. Such experiments would provide additional information on cell-cell communication in the expanding population, which was suggested to impact MSC expansion [36]. As already mentioned above, the tracking of MSC involves particular problems. Long term monitoring of MSC fates will require therefore sophisticated marker systems for both the clonal origin and the differentiation state of the cells. A number of stem cell and differentiation markers of MSC have been suggested. Good candidates are early transcription factors [37,38].

Long-term fluctuations in differentiation marker expression in single cells would directly proof our concept of noise-driven stem cell organisation. For the generality of our concept, we expect such fluctuations to underlie somatic stem cell organisation independent of tissue and species.

The impact of these fluctuations may vary between different stem cell systems according to functional requirements [39]. Thus, individual stem cell systems may appear as more or less hierarchical organised. The MSC system may exhibit a pronounced flexibility, in order to be capable of instantaneous fate decisions in the course of development and in case of injury [39,40].

## Conclusion

Understanding single cell behaviour is prerequisite to unveil general principles of the organisation of stem cell populations. Stem cell maintenance, expansion and environmental adaptation may in particular rely on single cell plasticity. Currently only limited data on the *in vitro *plasticity of individual stem cells are available. We here presented for the first time quantitative simulation results on *in vitro *MSC plasticity applying our novel concept of noise driven stem cell differentiation. Thereby we demonstrate the suitability of the IBM approach for studying these phenomena. Challenging current views on stem cell organisation, our results predict a highly dynamic stem cell pool, whose maintenance involves permanent de-differentiation events.

## Authors' contributions

AK performed all IBM simulations and following data processing. MH contributed the master equation approach, DD provided important assistance concerning IBM and ML concerning stem cells. JG did most of the intellectual contributions and major parts of draft writing. All authors have read and approved the final version of the manuscript.
